# Pathogenesis and Transmission of a Reassorted H1 Influenza A Virus Detected in North American Swine

**DOI:** 10.1111/irv.70228

**Published:** 2026-02-19

**Authors:** Débora B. Goulart, Carine K. Souza, Giovana C. Zanella, Celeste A. Snyder, Janice C. Zanella, Alexey Markin, Bailey Arruda, Tavis K. Anderson, Amy L. Baker

**Affiliations:** ^1^ National Animal Disease Center, Agricultural Research Service U.S. Department of Agriculture Ames Iowa USA; ^2^ Department of Veterinary Diagnostic and Production Animal Medicine Iowa State University Ames Iowa USA; ^3^ Embrapa Swine and Poultry, Brazilian Agricultural Research Corporation Concórdia Santa Catarina Brazil

**Keywords:** influenza virus, pandemic, pathogenesis, reassortment, swine, transmission, zoonosis

## Abstract

**Background:**

The USDA influenza A virus in swine national surveillance plan identified an increase in the detection frequency of a group of swine 1A.1.1.3 hemagglutinin (HA) clade viruses. This change was associated with reassortment events that resulted in new neuraminidase (NA) gene pairings. We hypothesized that the new N1 genes improved the transmission efficiency of the virus.

**Methods:**

We assessed the pathogenesis and transmission of four H1 1A.1.1.3 swine strains paired with different NA subtypes and lineages, all of which shared similar internal gene constellations.

**Results:**

There was little variation in the titers of viral nasal shedding across the groups, and the different surface protein pairings had no effect on transmission efficiency. All IAV strains, reflecting both pre‐ and post‐reassortment genetic patterns, were transmitted to naïve indirect contact pigs.

**Conclusions:**

These data indicate that the combinations of 1A.1.1.3 HA with NA subtypes had little impact on transmission. These findings suggest that the increased detection of the H1 1A.1.1.3 clade in the United States was unlikely directly due to altered replication, transmission, or antigenic drift, but perhaps due to changes in population immunity resulting from differential vaccine use or prior exposure, variations in production practices, or ecological conditions.

## 1 Introduction

Influenza A virus (IAV) causes illness ranging from asymptomatic infection to severe pneumonia and death in various domestic and wild animal species and humans [1]. Three IAV subtypes, H1N1, H1N2, and H3N2, are endemic in swine populations worldwide, with significant diversity in the hemagglutinin (HA), neuraminidase (NA), and the remaining six gene segments [2]. The diversity of swine IAVs presents a significant obstacle to pandemic risk assessment and control of the virus in swine. A primary reason for this diversity is that pigs are natural hosts to swine‐adapted strains and may also be permissive for avian‐ and human‐origin IAVs [3]. The observed genetic and antigenic diversity reflects the dynamic evolutionary path of endemic swine IAVs, driven by reassortment with avian‐ and human‐origin IAVs introduced into swine over the past century [4]. This diversity contributes to zoonotic transmission to humans and increases the risk of a second swine‐origin pandemic [5, 6, 7].

There are two IAV lineages within the H1 subtype that cocirculate in the United States [8]. These include the 1A classical swine lineage, derived from the 1918 human pandemic virus, which has been circulating in swine populations for over a century [9], and the 1B human seasonal lineage, derived from pre‐2009 human seasonal spillovers that became established in swine populations [8]. The 1A lineage includes the 1A.3.3.2 genetic clade that caused the 2009 H1N1 pandemic (H1N1pdm09) [10] and, along with four additional genetic clades (1A.1.1.3, 1A.4, 1A.3.3.2, and 1A.3.3.3), represents nearly 45.6% of all public IAV detections in US swine between 2022 and 2025 [11]. Swine H1 viruses may be paired with NA genes of either the N1 or N2 subtype, both of which reflect interspecies spillovers and subsequent transmission and evolution in swine. Within the N1 subtype, there are genes that share ancestry with the 1918 H1N1 virus (N1.classical) or genes that are associated with the 2009 H1N1 pandemic virus (N1.pdm) [12]. The N2 subtype detected in swine is associated with different H1N2 or H3N2 human‐to‐swine spillover events that have persisted and reassorted with endemic swine H1 viruses; these N2 genes are grouped by their year of introduction into swine (N2.1998, N2.2002, and N2.2016) [13, 14]. These endemic swine IAV surface protein genes are paired with six other genes that have genetic diversity reflected by interspecies transmission from birds and humans to swine and onward transmission in pigs following reassortment. These genes are categorized using an evolutionary lineage: TRIG (T), representing the triple‐reassortant virus detected in the 1990s [15]; H1N1pandemic (P), reflecting genes associated with the 2009 H1N1 pandemic virus [16]; and a live attenuated influenza virus vaccine briefly used in the United States [17]. Over the past decade, 29 different internal gene constellations have been detected in U.S. swine. Within the H1 subtype alone, 24 patterns have been identified, with one genome constellation, TTTPPT, representing 52.6% of all swine IAV detections [11]. Although few studies quantify the impact of the internal genes on virus phenotype [18, 19], evidence suggests that different gene pairings affect transmission efficiency and that reassortment in these genes spurs diversification of surface proteins.

The detection frequency of the H1N1 and H1N2 genetic groups of viruses in the United States varies annually, but the 1A.1.1.3 clade detection has increased in recent years, reaching 14.2% in 2022–2023 and then 21.4% in 2023–2024 [11]. Moreover, clade 1A.1.1.3 was the most frequently detected HA clade in the United States in 2023 [11]. The increase in 1A.1.1.3 detection frequency in 2021 coincided with multiple reassortment events, primarily involving the NA gene [11]. Prior to 2021, the 1A.1.1.3 HA gene was primarily paired with an N2 subtype gene from the N2‐2002B lineage [11]. However, between 2022 and 2024, the H1 1A.1.1.3 HA clade was increasingly paired with N1‐classical and N1‐pdm genes, and its detection frequency rose substantially [11]. For example, the frequency of the 1A.1.1.3/N1‐classical pairing increased by 33% from January 2022 to January 2024 [11]. During the same interval, the 1A.1.1.3/N1‐pdm pairing increased by 2.4%, and pairing with N2‐2002B rose by 2.8% [11]. These HA–NA gene pairings tended to maintain the same six internal genes.

In this study, we hypothesized that the increase in the detection frequency of the 1A.1.1.3 clade was driven by the acquisition of new NA genes that generated novel viruses with increased IAV transmission efficiency in swine. The NA gene of IAV enhances transmission efficacy by enzymatically cleaving mucins to increase contact with the respiratory epithelium [20, 21] and by removing immature virions from the surface of infected cells through destruction of sialic acid receptors [22, 23]. Additionally, immunity to the NA protein may contribute to protection [24], and antigenically novel NA genes could influence detection frequencies by reducing the protective impact of prior exposure or vaccination [12]. We evaluated representative strains from four distinct HA–NA gene pairings among the 1A.1.1.3 IAVs that circulated in 2022–2023, including the historically dominant N2‐2002B pairing, in a swine pathogenesis and transmission study. We demonstrated that different pairings of 1A.1.1.3 HA with different NA subtypes did not affect transmission efficiency. Although there was variation in the titers of viral nasal shedding across the groups, all IAV strains were transmitted to the indirect contact pigs. Our data suggest that reassortment to acquire new NA genes unlikely affected detection frequency of the 1A.1.1.3 due to transmission phenotype. We suggest that the increase in 1A.1.1.3 may instead be determined by the antigenic novelty of the N1 genes, the host immune landscape driven by prior exposure or vaccination, or agricultural practices that move pigs and viruses across the landscape, allowing for random reassortment events and viral dispersion. These data demonstrate that although reassortment alters genetic diversity, there was not a simple association between reassortment and transmission efficiency [25]. In conclusion, our findings highlight the relevance of characterizing reassorted IAVs to understand the viral and host factors that influence transmission dynamics and detection patterns in North American swine populations.

## 2 Materials and Methods

### 2.1 Genetic Analysis and Strain Selection

We used the octoFLUdb database (https://github.com/flu‐crew/octofludb) to query and classify all genetic sequences collected as part of the USDA IAV in swine surveillance system. This provided evolutionary lineage or clade classification for the HA and NA clades, as well as internal gene constellations using the octoFLU classifier [26]. We extracted HA and NA sequences associated with the H1 clade 1A.1.1.3, resulting in *n* = 593 HA–NA pairs collected between January 1, 2013, and May 31, 2023. The extracted HA and NA sequences were aligned separately using MAFFT v7.475 [27], and phylogenetic trees were inferred using FastTree v2.1.11 [28] under the generalized time‐reversible (GTR) model of nucleotide substitution with 20 gamma‐distributed rate categories [29]. We analyzed the reassortment history between the HA and NA gene segments among the 593 strains using TreeSort v0.1.6 [30]. To select representative 1A.1.1.3 HA–NA gene pairings, we used PARNAS v0.1.6 [31] with the HA phylogeny as input. We selected one representative strain that included a N2‐2002B to reflect the 1A.1.1.3 clade prior to reassortment and three post‐reassortment representative strains: one 1A.1.1.3 paired with an N1‐classical gene segment and two 1A.1.1.3 representatives paired with N1 genes derived from the 2009 H1N1 pandemic lineage (pdm09). An N1‐pdm09 representative A/swine/Missouri/A02861328/2023 H1N1 was a field isolate with reports of notable clinical disease (Phillip Gauger, personal communication). All selected strains were collected in 2022 and 2023 and shared a conserved internal genome constellation of TTTPPT. This genome constellation reflects different evolutionary lineages and is presented in the order of PB2‐PB1‐PA‐NP‐M‐NS, with each letter representing either triple reassortant origin (T) or H1N1 pandemic origin (P) genes. TTTPPT was the dominant internal gene constellation among 1A.1.1.3 clade viruses with whole‐genome sequence data collected between January 1, 2013, and May 31, 2023.

### 2.2 Viruses

The representative H1 1A.1.1.3 IAV isolates were obtained from the National Veterinary Services Laboratories (NVSL). The viruses used in this study were from the following HA and NA clades: 1A.1.1.3/N2‐2002B (A/swine/Ohio/A02750994/2022 H1N2), 1A.1.1.3/N1‐classical (A/swine/Indiana/A02750630/2022 H1N1), and 1A.1.1.3/N1‐pdm (A/swine/Missouri/A02750646/2022 H1N1 and A/swine/Missouri/A02861328/2023 H1N1). The viruses were propagated in Madin‐Darby canine kidney (MDCK) cells grown in Opti‐MEM (Life Technologies, Waltham, MA, USA). The virus growth media contained antibiotics and antimycotics and 1 μg/mL of tosyl phenylalanyl chloromethyl ketone (TPCK)‐trypsin (Worthington Biochemical Corp., Lakewood, NJ, USA). The NA activity of the viruses was assessed using an enzyme‐linked lectin assay (ELLA) [32], and results were expressed as EC_50_ values, representing the virus dilution that produced 50% of maximal NA activity in the linear range. Briefly, high‐binding 96‐well plates were coated with fetuin (Sigma, St. Louis, MO, USA) to allow binding. Viruses were diluted to a starting titer of 10^6^ TCID_50_ per mL, and twofold serial dilutions were prepared in supplemented Dulbecco's phosphate‐buffered saline (PBS). Diluted viruses were added to the coated plates and incubated for 16–18 h at 37°C. After incubation, plates were washed to remove unbound virus. Peanut agglutinin (PNA) (Sigma‐Aldrich, St. Louis, MO) conjugated to horseradish peroxidase (HRPO, Sigma) was added to detect exposed sialic acids released by NA activity. Following incubation and washing, o‐phenylenediamine dihydrochloride (OPD, Sigma) was added to generate a colorimetric signal. The reaction was stopped after 10 min with 1 N H_2_SO_4_, and absorbance was read at 650 nm. ELLA results were plotted as the log_2_ of the virus dilution divided by 10 on the x‐axis and the optical density (OD) on the y‐axis to calculate EC_50_, which was then reverse transformed to a reciprocal titer. Hemagglutination activity was assessed to determine the viral HA units. Viruses diluted to 10^6^ TCID_50_/mL were added to a V‐bottom plate and serially twofold diluted. A 0.5% red blood cell (RBC) suspension was added to each well. After 30‐min incubation at room temperature, the highest dilution showing visible agglutination was recorded as the HA titer for standardized comparison across all virus samples in this assay. Each virus was tested in duplicate in a biosafety level 2 (BSL‐2) laboratory.

### 2.3 Swine‐to‐Swine Transmission Study Design

Sixty‐five weaned, healthy pigs of mixed sex were confined in an ABSL‐2 containment facility at the National Animal Disease Center (NADC) in accordance with an approved US Department of Agriculture (USDA)—Agricultural Research Service (ARS) National Animal Disease Center (NADC) Institutional Animal Care and Use Committee Protocol. Pigs were confirmed to be free of porcine reproductive and respiratory syndrome virus (PRRSV) and of IAV antibodies. Before transport, pigs received prophylactic treatment with ceftiofur (Excede; Zoetis, Florham Park, NJ, USA) at the source farm, in accordance with label dosage, and received enrofloxacin (Baytril; Bayer Animal Health, Shawnee Mission, KS, USA) after arrival to resolve or prevent bacterial infections. Prior to the start of the study, serum was collected and tested for antibodies against influenza A nucleoprotein (NP) by a commercial enzyme‐linked immunosorbent assay (ELISA) kit (AI MultiS‐Screen kit, IDEXX, Westbrook, ME, USA) to confirm the absence of preexisting immunity from previous exposure or passively acquired maternal antibodies. Pigs were randomly assigned to four experimentally inoculated groups (*n* = 10) and one naïve control group (*n* = 5). Each animal was given a unique identification number and placed in separate confinement rooms. Pigs received a subcutaneous radio‐frequency microchip (Deston Fearing, Dallas, TX, USA) for daily body temperature monitoring, with readings recorded prior to IAV exposure for establishing a baseline.

**FIGURE 1:**
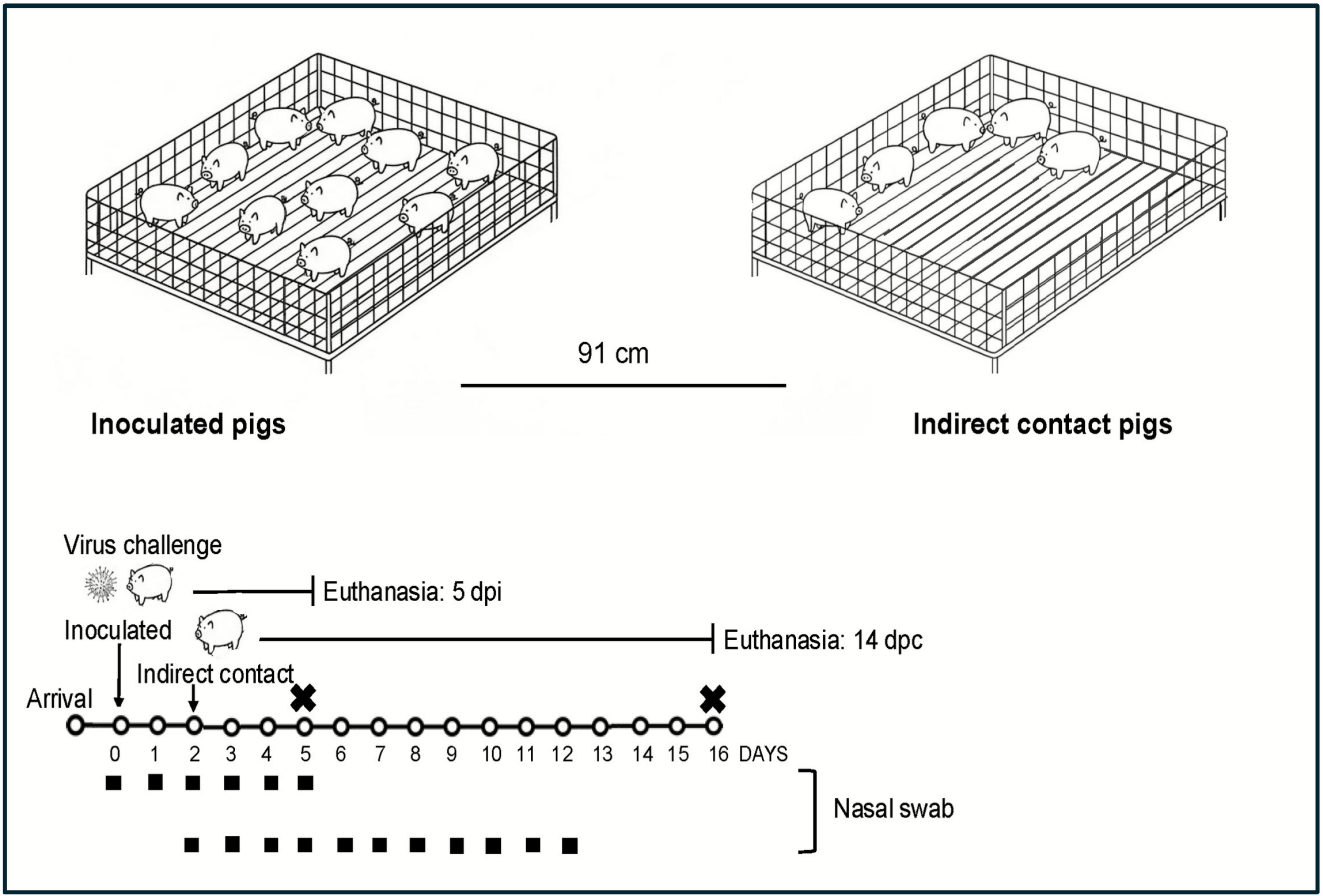
Diagram of study design. Ten pigs per group were inoculated intranasally and intratracheally with each assigned virus. Five indirect contact pigs were housed in a deck separated by approximately 91 cm within the same room of each inoculated group at 2 DPI. Timeline of sample collection and necropsy is shown.

Primary pigs were inoculated intranasally (1 mL) and intratracheally (2 mL) with 10^5^ 50% tissue culture infectious dose (TCID_50_)/mL of each assigned virus diluted in PBS in a separate ABSL‐2 containment room per virus group [23]. Inoculation was performed under sedation using an intramuscular injection of a cocktail consisting of ketamine (8 mg/kg of body weight), xylazine (4 mg/kg), and Telazol (6 mg/kg) (Zoetis Animal Health, Parsippany, NJ, USA). At 2 days post inoculation (DPI), five indirect contact pigs were placed in a separate deck approximately 91 cm from the deck housing the inoculated group in each ABSL‐2 containment room to evaluate indirect respiratory (airborne) transmission. Pigs were observed daily for respiratory clinical signs. To assess virus replication, nasal swab (NS) (FLOQSwabs, Copan Diagnostics, Murrieta, CA, USA) samples were collected from 0 to 5 DPI for inoculated pigs and from 0 to 5 days post contact (DPC), then on 7 and 9 DPC for indirect contact pigs, following methods previously described [33]. To evaluate lung lesions, inoculated pigs were then humanely euthanized with a lethal dose of pentobarbital (Fatal‐Plus, Vortech Pharmaceuticals, Dearborn, MI, USA) and necropsied at 5 DPI, as previously described [34]. Postmortem samples included bronchoalveolar fluid (BALF), trachea, and right cardiac or affected lung lobe. Indirect contact pigs were humanely euthanized at 14 DPC. Figure 1 shows a diagram of the study design.

To confirm indirect transmission to contact pigs, sera from indirect contact pigs collected at 14 DPC were analyzed by hemagglutination‐inhibition (HI) assay and ELISA. Prior to the HI assay, sera were treated with a 20% Kaolin suspension (Sigma‐Aldrich, St. Louis, MO, USA), heat‐inactivated at 56°C, and adsorbed with 50% turkey RBC using standard techniques [35]. Reciprocal titers were divided by 10‐fold, log_2_‐transformed, and reported as the geometric mean. To assess antigenic relationships among the four viruses, cross‐HI assays were performed. Sera from five pigs from each of the four virus exposure groups were tested against all four viruses. Antigenic similarity was inferred when the difference in HI titer between homologous and heterologous virus–antiserum pairs was less than fourfold.

### 2.4 Virus Replication and Shedding

NS and BALF samples were titrated on confluent MDCK cells to assess viral replication in the nasal cavities and lungs, as previously established [34]. The cytopathic effect (CPE) of MDCK‐inoculated monolayers was assessed 48–72 h post‐infection. Monolayers were fixed using 4% phosphate‐buffered formalin and stained via immunocytochemistry (ICC) with an anti‐influenza A NP monoclonal antibody [36]. Virus titers (TCID_50_/mL) were calculated for each sample according to the Reed and Muench method [37]. NS samples that tested positive by virus isolation but negative by virus titration were confirmed using the qPCR VetMAX‐Gold SIV Detection Kit (Thermo Fisher Scientific, MA, USA). Samples were analyzed by qPCR with a standard curve ranging from 10 to 1,000,000 copies per μL. The detection limit for virus titration was 0.5 log_10_ TCID_50_/mL.

### 2.5 Pathological Examination

The percentage of the lung affected by the purple‐red consolidation typical of IAV was assessed after the lungs were removed during necropsy at 5 DPI [34]. The percentage of pneumonia on the lung surface was visually evaluated for each lobe. Weighted proportions of each lobe relative to total lung capacity were used to compute the total percentage of lung involvement [38]. Tissue samples from the trachea and lungs were fixed in 10% buffered formalin for histopathologic analysis and stained with hematoxylin and eosin (H&E). Using previously established criteria [39], a veterinary pathologist, blinded to treatment groups, evaluated microscopic tracheal lesions and assigned a combined tracheal lesion score on a scale of 0–8. The same veterinary pathologist evaluated microscopic lung lesions and assigned a combined lung lesion score on a scale of 0–20 [40].

### 2.6 Microbiological Assays

BALF samples were screened for aerobic bacterial growth on blood agar and Casmin (NAD‐enriched) plates to detect possible coexistence of bacterial pneumonia. Samples were screened for other respiratory pathogens, such as PRRSV, porcine circovirus types 2 and 3 (PCV2 and PCV3), and *Mycoplasma hyopneumoniae* (VetMax, Life Technologies, Carlsbad, CA, USA) by qPCR [20] following the guidelines provided by the manufacturer.

### 2.7 Data Analysis

Statistical analysis to compare different treatment groups was performed using analysis of variance (ANOVA) with a significance level of *p* < 0.05 using Graph Pad Prism 8 Software (GraphPad, San Diego, CA, USA). Variables that exhibited significant differences among treatment groups were further analyzed through pairwise mean comparisons using the Tukey–Kramer test. Reciprocal HI titers were converted to a log2 scale, geometric means were calculated, and means transformed back to reciprocal geometric mean titers. Neuraminidase EC_50_ values for each virus were determined using a Log(agonist) versus response curve in Graph Pad Prism 8, with the log_2_ virus dilution plotted on the x‐axis and the OD on the y‐axis. EC_50_ titers were extrapolated from the resulting dose‐response curve for each virus.

## 3 Results

### 3.1 H1 1A.1.1.3 Reassortment and Genomic Evolution

**FIGURE 2:**
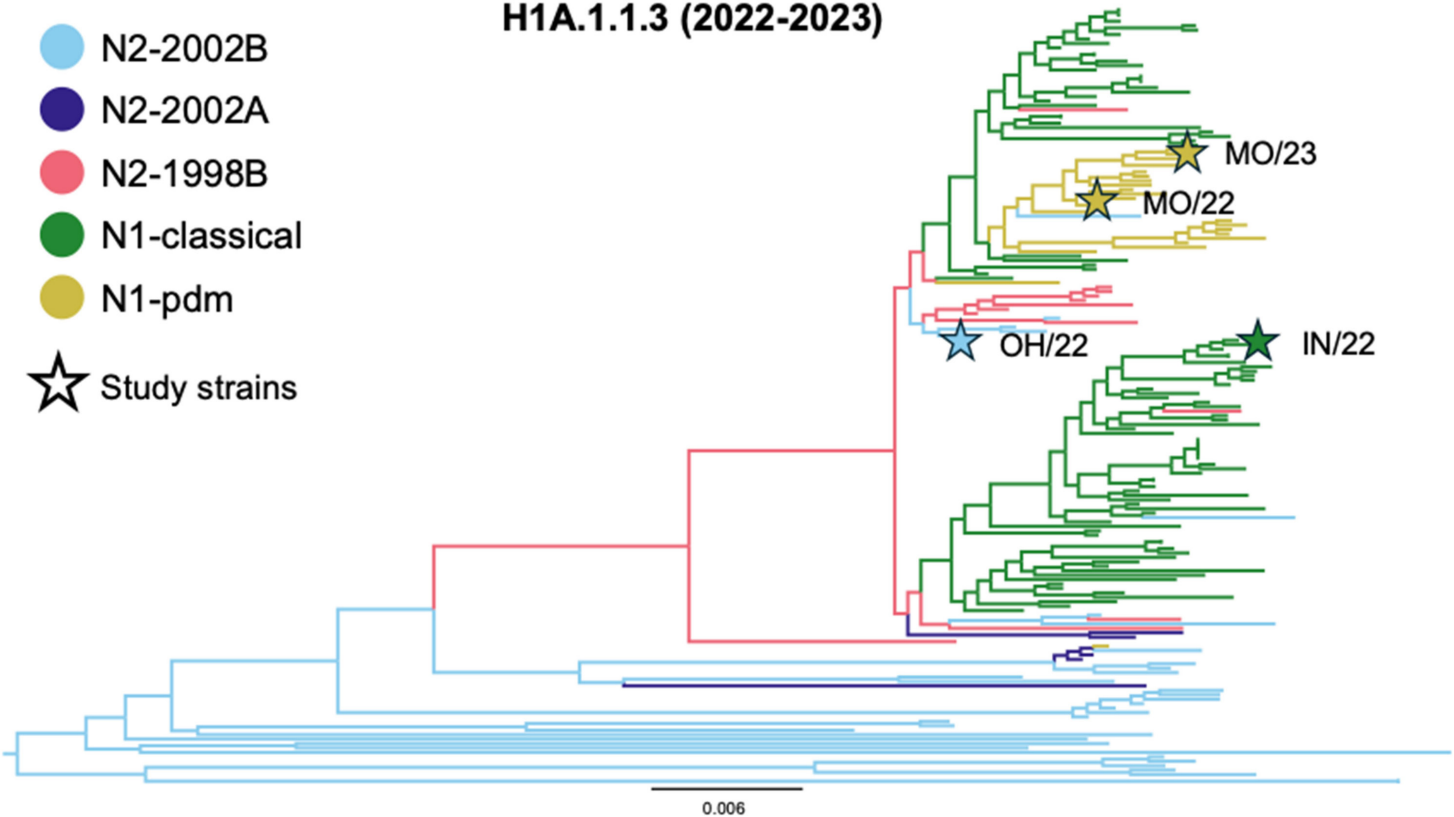
Evolutionary history of H1 subtype influenza A virus in swine from the 1A.1.1.3 hemagglutinin group collected in the United States between January 2022 and May 2023. The branches of the tree were colored by the associated NA clade assigned by smot v0.16.0 [42]. The strains selected for this study were annotated on the tree by stars with the color of the star reflecting the evolutionary lineage of the neuraminidase gene. Stars indicate the following viruses: MO/23 1A.1.1.3/N1‐pdm (A/swine/Missouri/A02861328/2023 H1N1), MO/22 1A.1.1.3/N1‐pdm (A/swine/Missouri/A02750646/2022 H1N1), OH/22 1A.1.1.3/N2‐2002B (A/swine/Ohio/A02750994/2022 H1N2), and IN/22 1A.1.1.3/N1‐classical (A/swine/Indiana/A02750630/2022 H1N1). The selected strains represent three distinct NA clades that were most frequently associated with 1A.1.1.3 hemagglutinin across this time period.

Among the swine IAV strains collected between January 2022 and May 2023, the 1A.1.1.3 HA gene was paired with five different NA clades, including three distinct N2 clades (1998B, 2002A, and 2002B) [41] and two N1 clades (N1.classical swine lineage and N1.pdm09) [12]. We identified 17 reassortment events involving the 1A.1.1.3 HA switching to a different NA clade (Figure 2). The estimated HA–NA reassortment rate was 0.073 events per year per virus, which is approximately 2.5 times higher than the general HA–NA reassortment rate for swine H1s previously reported [30]. Previously, the 1A.1.1.3 HA was predominantly paired with N2 genes [11], whereas a shift occurred in the 2022–2023 time period, and the N1 gene is now the most frequently detected pairing with this HA [43]. To understand whether this shift was linked to changes in virus phenotype, we selected four representative strains, one representing the previously dominant H1N2 subtype and three representing H1N1 strains with two different N1 lineages (Figure 2). The four viruses exhibited NA activity within a twofold difference when standardized to a starting titer of 10^6^ TCID_50_/mL, with N2‐2002B exhibiting an EC_50_ reciprocal titer of 275, followed by N1‐classical with 266, N1‐pdm‐MO/23 with 164, and N1‐pdm‐MO/22 with 152. All four viruses also displayed similar HA activity with N2‐2002B and N1‐classical reciprocal titers of 256 HA units and N1‐pdm‐MO/22 and N1‐pdm‐MO/23 reciprocal titers of 128 HA units.

### 3.2 Lower Respiratory Tract Pathology and Viral Detection in the Lungs of Inoculated Pigs

**FIGURE 3:**
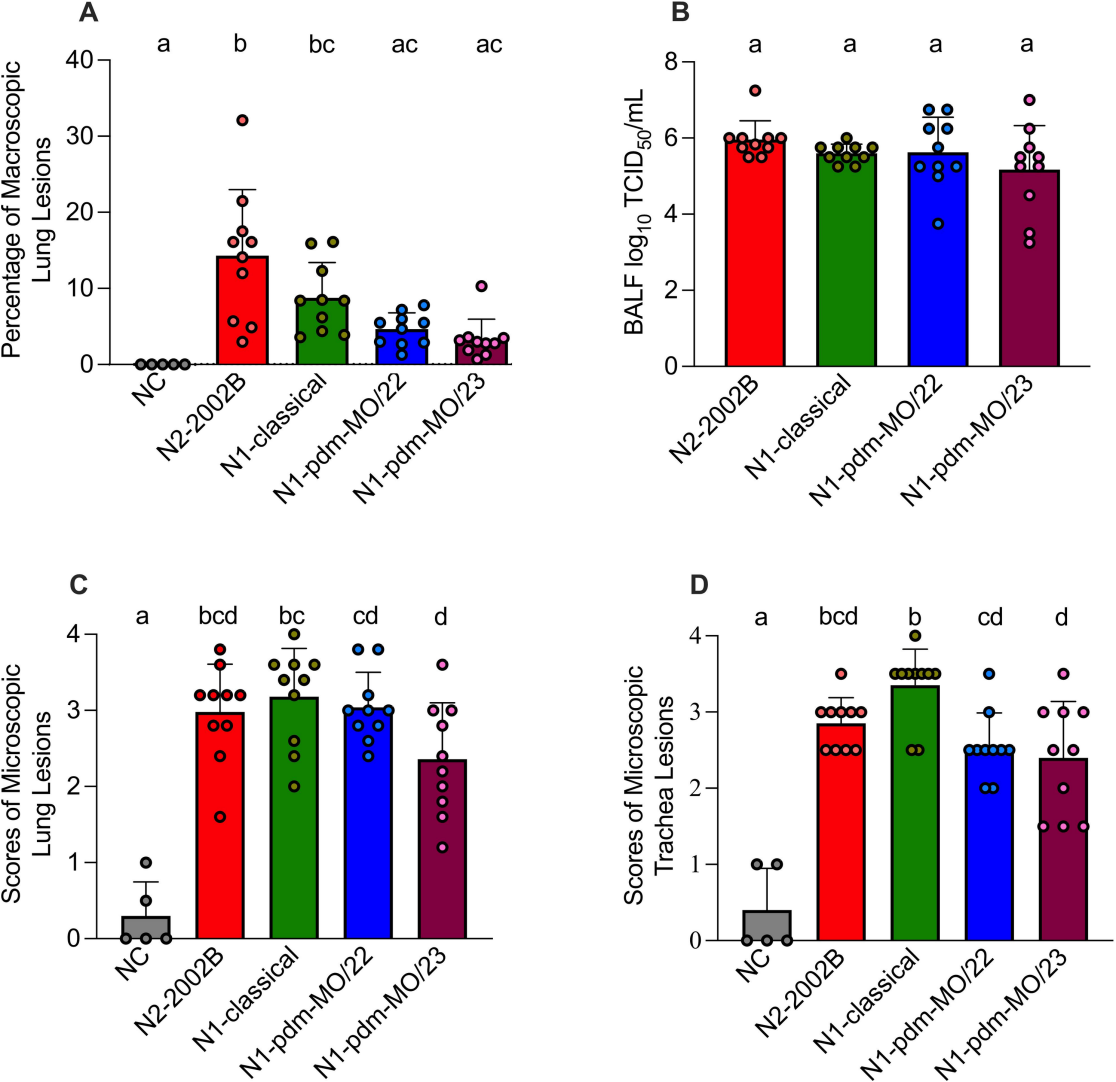
Macroscopic lung lesions, viral titers in BALF, and scores of microscopic lung and tracheal lesions in pigs inoculated with 1A.1.1.3 representative strains paired with N2‐2002B, N1‐classical, N1‐pdm‐MO/22, or N1‐pdm‐MO/23 at 5 DPI. (A) Percentage of lung affected with purple‐red consolidation; (B) viral titers in BALF; (C) scores of microscopic lung lesions (0–20); and (D) scores of microscopic tracheal lesions (0–8). Data are presented as mean ± standard error of the mean (SEM), with individual pigs represented by scattered dots. Bars that do not share lowercase letters indicate statistically significant differences between group means at *p* < 0.05. NC, negative control.

The percentage of macroscopic lung lesions, viral titers in BALF, and microscopic lung and tracheal lesion scores in inoculated pigs at 5 DPI are presented in Figure 3. The 1A.1.1.3/N2‐2002B virus induced a significantly higher percentage of macroscopic lung lesions in pigs (14.3 ± 8.7) compared to all other groups, except for N1‐classical (8.8 ± 4.6) (Figure 3A). The average percentage of macroscopic lesions was low in pigs inoculated with 1A.1.1.3 paired with N1‐pdm‐MO/22 (4.7 ± 2.1) or N1‐pdm‐MO/23 (3.3 ± 2.6), and these values were not statistically different from the NC group. All challenged groups exhibited similar high virus titers in the lungs, with no significant differences between groups (Figure 3B). Pigs inoculated with 1A.1.1.3/N1‐classical, 1A.1.1.3/N1‐pdm‐MO/22, or 1A.1.1.3/N2‐2002B exhibited moderate microscopic lung lesions, whereas those inoculated with N1‐pdm‐MO/23 presented mild lesions (Figure 3C). Pigs inoculated with A.1.1.3 paired with either N1‐classical or N2‐2002B showed moderate microscopic tracheal lesions, whereas pairing with N1‐pdm‐MO/22 or N1‐pdm‐MO/23 resulted in mild lesions (Figure 3D).

### 3.3 Nasal Virus Shedding in Inoculated Pigs

**FIGURE 4:**
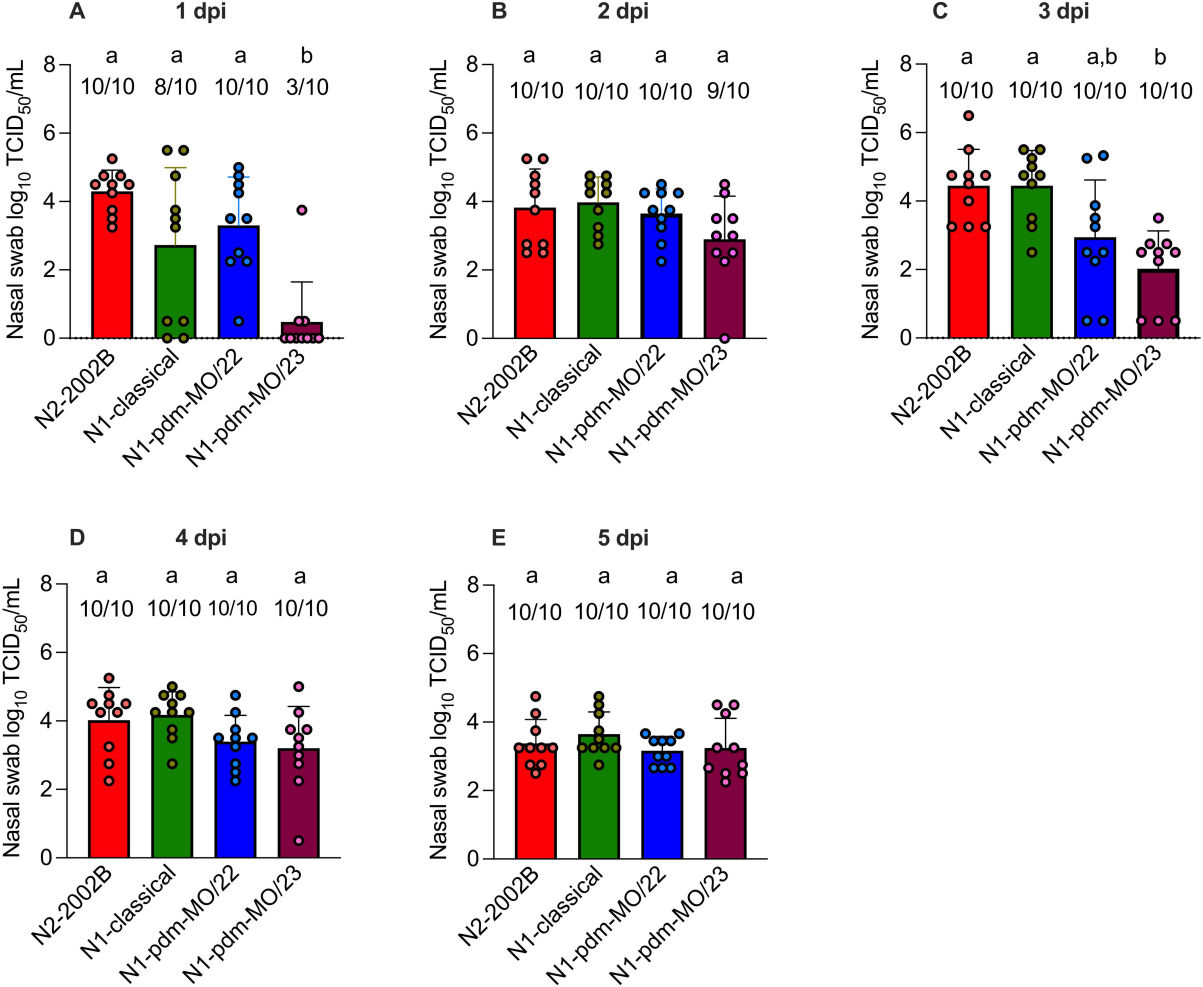
Viral titers in nasal swabs from inoculated pigs collected from 1 to 5 DPI with IAVs. (A–E) Data from 1 to 5 DPI, respectively. Numbers above the error bars indicate the number of positive pigs out of the total number in each group. Bars that do not share lowercase letters indicate statistically significant differences between group means at *p* < 0.05. Values are presented as mean log_10_ TCID_50_/mL titers ± SEM.

All pigs inoculated with 1A.1.1.3/N2‐2002B shed virus at all time points, with group mean titers of 4.3, 3.8, 4.5, 4.0, and 3.4 log_10_ TCID_50_/mL on 1, 2, 3, 4, and 5 DPI, respectively (Figure 4). Similarly, all pigs inoculated with 1A.1.1.3/N1‐pdm‐MO/22 shed virus at every time point, with group mean titers of 3.3, 3.7, 2.9, 3.4, and 3.2 log_10_ on 1–5 DPI, respectively. In the 1A.1.1.3/N1‐classical group, two pigs did not shed virus on 1 DPI; however, all pigs shed virus from 2 to 5 DPI, with group mean titers of 4.0, 4.5, 4.2, and 3.7 log_10_ on 2–5 DPI, respectively. Three pigs inoculated with 1A.1.1.3/N1‐pdm‐MO/23 shed virus on 1 DPI, resulting in a low group mean titer of 0.5 log_10_. On 2 DPI, nine out of 10 pigs shed virus, and all pigs shed virus from 3 to 5 DPI, with group mean titers of 2.9, 2.0, 3.2, and 3.2 log_10_, respectively. There were no significant differences in viral shedding among the four groups on 2, 4, and 5 DPI. However, because only three pigs shed N1‐pdm‐MO/23 on 1 DPI, the group mean titer for this group was significantly lower than that of the other groups (0.5 log_10_ vs. a combined average of 3.4 log_10_). Similarly, on 3 DPI, the N1‐pdm‐MO/23 group had a significantly lower group mean titer compared to the N2‐2002B and N1‐classical groups (2.0 log_10_ vs. a combined average of 4.5 log_10_) but was not statistically different from the N1‐pdm‐MO/22 group (2.9 log_10_).

### 3.4 Transmission to Indirect Contact Pigs

**FIGURE 5:**
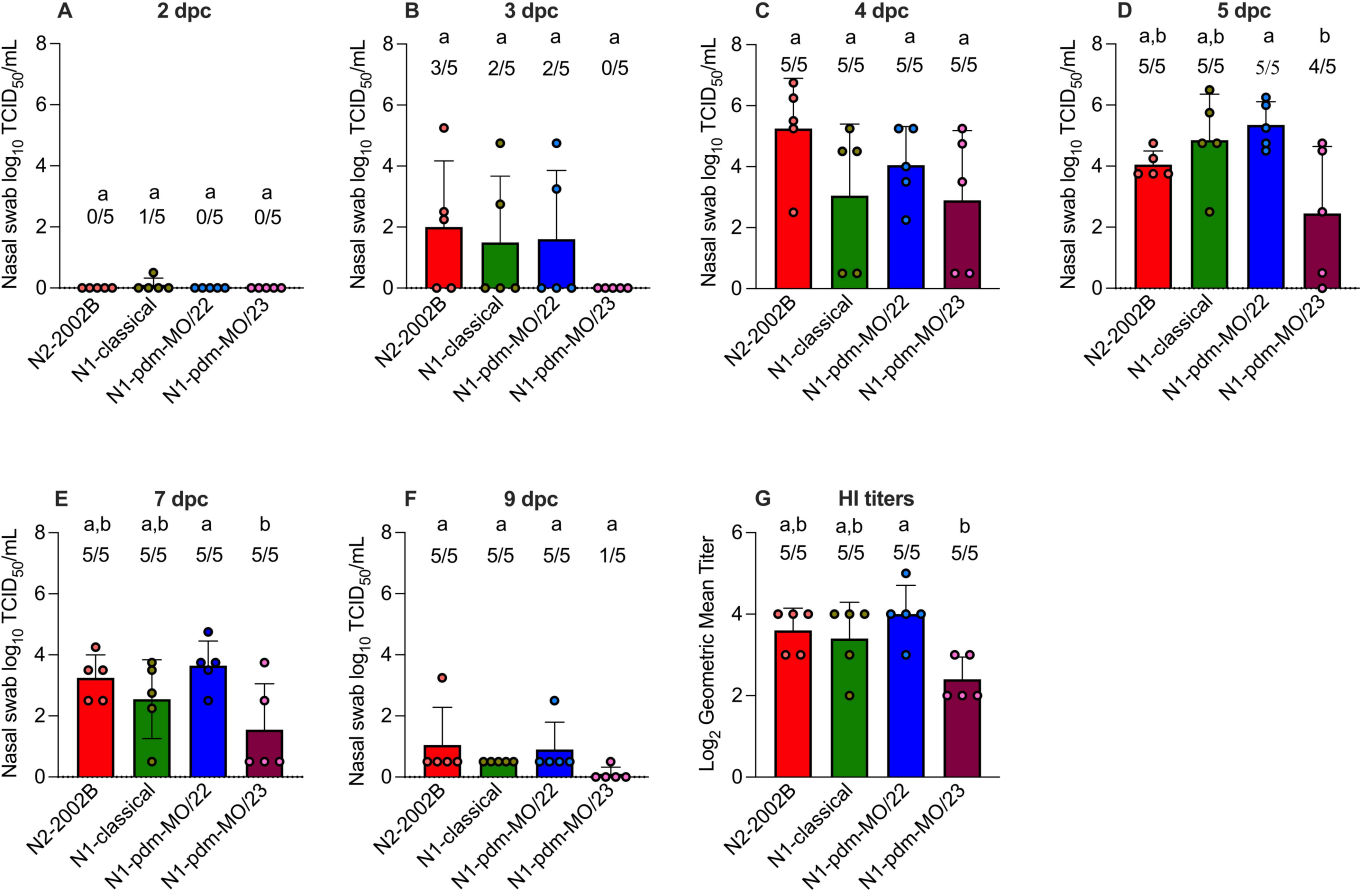
Evidence of transmission in indirect contact pigs. (A–F) Viral titers in nasal swabs of indirect contact pigs following infection with IAVs, measured from 2 to 5 DPC, then again at 7 and 9 DPC. Values are shown as mean log_10_ TCID_50_/mL titers ± SEM. Numbers above the error bars indicate the number of positive indirect contact pigs out of the total in each group. Different lowercase letters indicate significant difference at *p* < 0.05. (G) Antibody response in indirect contact pigs at 14 DPC, measured by HI assay. Values are shown as mean log_10_ geometric mean titers ± SEM. Numbers above the error bar depict the number of seropositive pigs out of the total in each group. Bars that do not share lowercase letters indicate statistically significant differences between group means at *p* < 0.05.

NS viral titers were assessed in indirect contact pigs from 0 to 5 DPC, as well as at 7 and 9 DPC (Figure 5). No virus shedding was detected on 0 or 1 DPC, and these time points are not shown. The three viruses displayed different levels of transmissibility to indirect contact pigs. In the 1A.1.1.3/N1‐classical group, one out of five indirect contact pigs shed virus at 2 DPC with a titer of 0.5 log_10_ TCID_50_/mL; two out of five pigs shed virus at 3 DPC with an average titer of 1.5 log_10_; and all five pigs shed virus at 4 and 5 DPC, with an average titer of 3.1 log_10_ and 4.9 log_10_, respectively. Although all indirect contact pigs remained infected afterward, the average titer decreased markedly to 2.6 log_10_ at 7 DPC and 0.5 log_10_ at 9 DPC. In summary, at least one contact pig in the 1A.1.1.3/N1‐classical group was positive at every time point, totaling 23 positive samples throughout the experiment.

In the 1A.1.1.3/N2‐2002B group, three out of five indirect contact pigs shed virus at low levels at 3 DPC, with an average titer of 2 log_10_ TCID_50_/mL, and no virus shedding was detected at 2 DPC. All five indirect contact pigs shed virus at 4 DPC, with a peak average titer of 5.2 log_10_. The average titer declined gradually over the remainder of the study, reaching 4.1 log_10_ at 5 DPC, 3.3 log_10_ at 7 DPC, and 1.1 log_10_ at 9 DPC. Although titers decreased sharply, particularly at 9 DPC, all indirect contact pigs continued to shed virus at the final three time points. To summarize, at least three indirect contact pigs housed with 1A.1.1.3/N2‐2002B‐inoculated animals tested positive at every time point except 1 and 2 DPC, when no virus shedding was detected, totaling 23 positive samples.

In the 1A.1.1.3/N1‐pdm‐MO/22 group, two out of five indirect contact pigs shed virus at 3 DPC, with an average titer of 1.6 log_10_ TCID_50_/mL. All five indirect contact pigs shed virus at 4 DPC with an average titer of 4.1 log_10_, and viral titers increased at 5 DPC, reaching a peak of 5.4 log_10_. Although all indirect contact pigs shed virus until the end of the study, viral titers decreased significantly, dropping to 3.7 log_10_ at 7 DPC and 0.9 log_10_ at 9 DPC. None of the N1‐pdm‐MO/22 indirect contact pigs shed virus at 2 DPC. However, two indirect contact pigs began shedding at 3 DPC and continued through the end of the study, yielding a total of 22 positive samples.

All indirect contact pigs in the 1A.1.1.3/N1‐pdm‐MO/23 group shed virus at 4 DPC, with a peak average titer of 2.9 log_10_ TCID_50_/mL. The average titer then decreased to 2.5 log_10_ on 5 DPC, with four out of five indirect contact pigs shedding virus. Viral titers continued to decline on 7 DPC, with all indirect contact pigs testing positive at an average of 1.6 log_10_. By 9 DPC, the average titer was 0.1 log_10_, with only one pig shedding virus. None of the indirect contact pigs in the 1A.1.1.3/N1‐pdm‐MO/23 group shed virus on 2 or 3 DPC. However, most began shedding by 4 DPC and continued until the end of the study, resulting in a total of 15 positive samples.

All pigs from all indirect contact groups seroconverted by 14 DPC (Figure 5G). Antigenic phenotypes among the four challenge strains were assessed by HI assay and comparison between homologous and heterologous geometric mean titers of the five seroconverted indirect contact pigs in each group. The difference in HI titer between homologous and heterologous virus–antiserum pairs was less than fourfold, meaning that no significant antigenic differences were observed among the four viruses (Table 1).

**TABLE 1:** Antigenic relationships among the four viruses by hemagglutination inhibition assays.

Antigens	N2‐2002B^a^	N1‐classical	N1‐pdm‐MO/22	N1‐pdm‐MO/23
A/swine/Ohio/A02750994/2022	160	70	211	80
A/swine/Indiana/A02750630/2022	80	106	121	53
A/swine/Missouri/A02750646/2022	160	80	243	106
A/swine/Missouri/A02861328/2023	243	61	160	121

^a^ Antisera in columns from five indirect contact exposure pigs at 14 DPC. Results are shown in reciprocal geometric mean titers. Homologous titers are highlighted in gray.

## Discussion

Swine populations harbor a diversity of endemic IAVs, and cocirculation patterns reflect virus fitness, the host immune landscape, and the ecology of modern agricultural production. Evolutionary dynamics such as reassortment and immune‐driven antigenic drift result in viruses distinct from IAVs in human populations and represent a zoonotic threat [44, 45, 46]. A recent risk assessment of North American H1 IAVs in swine found almost no cross‐reactivity in human sera to the 1A.1.1.3 swine virus. This clade was also the most antigenically distant from the human vaccine strains [11, 46]. Understanding what factors affect the detection frequency of 1A.1.1.3 swine viruses in the United States can facilitate the control of the virus in swine populations and enhance pandemic preparedness and risk assessments. In this study, we evaluated the pathogenesis and transmission in pigs of the 1A.1.1.3 HA gene paired with an N2‐2002B, the most frequently detected NA pairing prior to reassortments detected in increasing frequency after 2021, and three post‐reassortment pairings: an N1‐classical and two N1 pandemic lineage genes derived from human seasonal introductions (pdm09‐classical human influenza viruses from the 2010s). Our data suggest that different pairings of the 1A.1.1.3 HA with N1 genes did not enhance IAV transmission in swine. For the 1A.1.1.3/N1‐pdm‐MO/23, transmission kinetics was negatively impacted by the N1pdm23 pairing. These findings suggest that the increased detection frequency of the H1 1A.1.1.3 clade was driven by other factors, such as population immunity arising from differential vaccine use and composition, previous exposure, or variation in ecological and production practices.

All IAV strains inoculated the pigs and were transmitted to the indirect contact pigs. Infection in contact pigs was confirmed by HI titers ranging from 40 to 320. In line with our findings, a previous study conducted by our group found that the 1A.1.1.3 swine virus achieved 100% transmission from pigs to ferrets, with all ferrets both shedding the virus and seroconverting [47]. However, one virus showed lower replication efficiency, 1A.1.1.3/N1‐pdm‐MO/23, compared to the other groups, as only one inoculated pig shed virus at 1 DPI (Figure 4A). This decreased viral shedding in the inoculated pigs likely delayed transmission to the indirect contact pigs, as pigs in this group began shedding virus 1 day later post‐contact than those in the other groups (Figures 5B and 4C). However, despite the decreased transmission kinetics, the 1A.1.1.3/N1‐pdm‐MO/23 strain was eventually transmitted to all indirect contact pigs, as confirmed by positive geometric mean titers (average of 2.4 log_2_) (Figure 5G). The 1A.1.1.3/N1‐pdm‐MO/23 strain reproduced to comparable levels in the lungs of inoculated pigs compared to other virus groups, suggesting a possible preference for the lower respiratory tract compared to nasal epithelium for this strain.

The other two 1A1.1.3/N1 viruses replicated in the upper respiratory tract and transmitted similarly to the 1A.1.1.3/N2‐02 strain (Figure 3). Although all viruses infected pigs and replicated efficiently in the lungs, 1A.1.1.3/N2‐2002B and N1‐classical strains produced more macroscopic lung lesions than the other groups (Figure 3). Previous investigations have reported a similar discordance between the percentage of lung lesions and viral replication in the lungs [48, 49]. The reason for the discordance remains unclear and requires further investigation, as several factors influence influenza pathogenesis, including induction of inflammation, infectivity, virulence, and replication efficiency—for example, the balance between HA binding and NA cleavage activities [50]. The NA and HA activity results were consistent with the viral titers in BALF, microscopic lung lesions, and tracheal lesion scores, all showing no significant differences among the viruses. However, N2‐2002B exhibited a higher macroscopic percentage of lung consolidation compared to N1‐pdm‐MO/22 and N1‐pdm‐MO/23, suggesting that N2‐2002B may induce greater inflammation, though these differences require further investigation. Specific gene segments, along with the combination and balance of multiple genes, collectively modulate influenza A viral fitness (e.g., replication and transmission) and phenotype [25]. A previous study used a human‐seasonal H3N2 virus and a swine H3N1 virus to assess the contributions of gene combinations to IAV replication and transmission in swine [7]. The study showed that a swine‐adapted HA gene alone enabled replication in the lungs, regardless of the NA or internal gene constellations of the two genotypes paired with it. However, efficient replication in the upper respiratory tract and transmission to indirect contact pigs depends on specific combinations with NA and/or internal genes. The glycoproteins HA and NA recognize sialic acid and exhibit complementary functions. HA binds sialic acid on host cells through its receptor‐binding site to initiate viral infection, whereas NA facilitates the release of new virions by cleaving sialic acid residues from the cell surface. The balance between HA (binding) and NA (cleavage) exhibits considerable flexibility, and its regulation has a greater impact on viral replication and interspecies transmission than either HA affinity or NA activity independently [51]. For example, viral release would be hampered if HA bound sialic acid with excessively high affinity or if NA were inactive, as seen when inhibitors were present [50]. Additionally, HA and NA continuously evolve through a series of amino acid substitutions. If these changes disrupt the existing HA–NA balance, they could lead to alterations in one or both glycoproteins, affecting viral fitness [52, 53].

Viruses with increased detection frequency should be investigated to understand how the interaction between HA and NA combinations impacts IAV transmission in swine. In our study, reassortment to acquire a new N1 gene did not explain the expansion of the 1A.1.1.3 HA clade in the swine population. The increased detection frequency of the H1 1A.1.1.3 clade is also unlikely to be due to antigenic drift, as the four strains were antigenically similar (based on cross‐HI data showing < 4‐fold changes). Instead, the rise in detection frequency of this clade may be attributed to external contributors focused on host and environmental factors, including variations in vaccine use and composition, prior exposure histories, and differences in production practices and ecological conditions. The TTTPPT constellation increased in detection frequency within the H1 1A.1.1.3 clade, rising from 22.2% in 2018–2019 to 27.6% in 2019–2020, becoming the most detected constellation in the H1 1A.1.1.3 clade for the first time [11]. In the following years, the TTTPPT constellation continued to increase in detection, comprising 83.3% of the detections within the H1 1A.1.1.3 clade in 2024–2025 [11]. When evaluating all IAV clades, the detection frequency of the TTTPPT constellation significantly increased from 24.6% in 2018–2019 to 75.8% in 2024–2025 [11]. Given the higher detection frequency of the TTTPPT constellation in the H1 1A.1.1.3 clade, this constellation might be associated with the increased fitness of the IAVs used in our study [25]. The characteristics necessary for a virus to spread effectively and establish itself in pig populations are largely unclear and most likely relate to the entire genome. Routine whole‐genome sequencing can help predict when an HA–NA combination will increase or decrease in detection frequency by identifying internal genes or constellations that have been shown to impact transmission or represent unique pairings.

Our study indicated that different pairings of 1A.1.1.3 HA with N1 subtype clades that all contained TTTPPT genotypes did not show a difference in pathogenesis or improve transmission efficiency over the N2‐02 pairing, as all three N1 viruses infected 10 primary pigs and were transmitted to five indirect contact pigs per group. Consequently, the increased detection frequency of the H1 1A.1.1.3 clade could be due to the immunological landscape of the swine population or variations in production methods and ecological conditions. Future research incorporating serologic analyses, such as HI assays using field sera, will be crucial to assess the importance of population immunity in driving the dynamics of 1A.1.1.3 virus detection. Moreover, given the higher detection frequency of the TTTPPT constellation currently present in the H1 1A.1.1.3 clade, it is plausible that this constellation might be associated with the increased fitness of the four HA–NA pairings in the strains used in our study in a similar manner observed with the constellation paired with an H3 subtype HA gene [25]. It is imperative to elucidate the mechanisms by which reassortment and evolutionary events give rise to more transmissible IAV strains and how these alterations influence the detection frequency of the H1 1A.1.1.3 clade. Understanding the mechanisms that facilitate the evolution and persistence of IAVs in swine populations is essential for the development of targeted health interventions aimed at mitigating viral transmission between pigs and from pigs to humans.

## Author Contributions

**Débora B. Goulart:** investigation (lead), formal analysis (lead), writing – original draft (lead). **Carine K. Souza:** formal analysis (supporting), writing – review and editing (equal). **Giovana C. Zanella:** formal analysis (supporting), writing – review and editing (equal). **Celeste A. Snyder:** formal analysis (supporting), writing – review and editing (equal). **Janice C. Zanella:** formal analysis (supporting), writing – review and editing (equal). **Alexey Markin:** investigation (equal), data curation (lead), writing – original draft (supporting). **Bailey Arruda:** formal analysis (supporting), supervision (equal), writing – review and editing (equal). **Tavis K. Anderson:** conceptualization (equal), supervision (equal), writing – review and editing (equal). **Amy L. Baker:** conceptualization (equal), funding acquisition (lead), investigation (equal), project administration (lead), resources (lead), supervision (lead), writing – review and editing (equal).

## Funding

This work was supported in part by the USDA‐ARS (ARS project number 5030‐32000‐231‐000D); USDA‐APHIS (ARS project numbers 5030‐32000‐231‐104‐I and 5030‐32000‐231‐111‐I); the National Institute of Allergy and Infectious Diseases, National Institutes of Health, Department of Health and Human Services (Contract No. 75N93021C00015); the Centers for Disease Control and Prevention (contract numbers 21FED2100395IPD and 24FED2400250IPC); and the SCINet project of the USDA‐ARS (ARS project number 0500‐00093‐001‐00‐D). Participants were supported in part by an appointment to the Agricultural Research Service (ARS) Research Participation Program administered by the Oak Ridge Institute for Science and Education (ORISE) through an interagency agreement between the US Department of Energy (DOE) and the US Department of Agriculture (USDA). ORISE is managed by ORAU under DOE contract number DE‐SC0014664 The funders had no role in study design, data collection and interpretation, or the decision to submit the work for publication.

## Ethics Statement

This study was approved by the USDA‐ARS National Animal Disease Center Institutional Animal Care and Use Committee.

## Conflicts of Interest

The authors declare no conflicts of interest.

## Data Availability

Clinical data associated with this study are available for download from the USDA Ag Data Commons at 10.15482/USDA.ADC/29991676. All genetic sequence data are available at NCBI GenBank with USDA IAV in swine surveillance data within a searchable interface at https://flu‐crew.org. The code associated with analysis in this article is provided at https://github.com/flu‐crew/datasets/tree/main/h1‐alphadel‐N1‐reassortment.
